# Novel biomarkers and age-related metabolite correlations in plasma and dried blood spots from patients with succinic semialdehyde dehydrogenase deficiency

**DOI:** 10.1186/s13023-020-01522-5

**Published:** 2020-09-23

**Authors:** Trevor Kirby, Dana C. Walters, Xutong Shi, Coleman Turgeon, Piero Rinaldo, Erland Arning, Paula Ashcraft, Teodoro Bottiglieri, Melissa DiBacco, Phillip L. Pearl, Jean-Baptiste Roullet, K. Michael Gibson

**Affiliations:** 1 Department of Pharmacotherapy, College of Pharmacy and Pharmaceutical Sciences Building Room 210C, Washington State University, 412 E. Spokane Falls Boulevard, Spokane, WA 99202-2131 USA; 2 Mayo Clinic, Department of Laboratory Medicine and Pathology, Rochester, MN USA; 3 Baylor Scott & White Research Institute, Institute of Metabolic Disease, Dallas, TX USA; 4 Department of Neurology, Pediatric Neurology, Harvard Medical School and Boston Children’s Hospital, Boston, USA

**Keywords:** Dried blood spots, Amino acids, Acylcarnitines, Creatinine, guanidinoacetate, Age-dependent correlations, γ-Hydroxybutyrate (GHB), γ-Aminobutyrate (GABA)

## Abstract

**Background:**

Previous work has identified age-related negative correlations for γ-hydroxybutyric acid (GHB) and γ-aminobutyric acid (GABA) in plasma of patients with succinic semialdehyde dehydrogenase deficiency (SSADHD). Using plasma and dried blood spots (DBS) collected in an ongoing natural history study, we tested the hypothesis that other biomarkers would follow a similar age-related negative correlation as seen for GHB/GABA. Samples (mixed sex) included: patients (*n* = 21 unique samples, 1–39.5 yrs) and parallel controls (*n* = 9 unique samples, 8.4–34.8 yrs). Archival control data (DBS only; *n* = 171, 0.5–39.9 yrs) was also included.

**Results:**

Metabolites assessed included amino acids (plasma, DBS) and acylcarnitines, creatine, creatinine, and guanidinoacetate (DBS only). Age-related negative correlations for glycine (plasma, DBS) and sarcosine (*N*-methylglycine, plasma) were detected, accompanied by elevated proline and decreased levels of succinylacetone, argininosuccinate, formaminoglutamate, and creatinine. Significantly low acylcarnitines were detected in patients across all chain lengths (short-, medium- and long-chain). Significant age-dependent positive correlations for selected acylcarnitines (C6-, C12DC(dicarboxylic)-, C16-, C16:1-, C18:1-, C18:2OH-carnitines) were detected in patients and absent in controls. Receiver operating characteristic (ROC) curves for all binary comparisons revealed argininosuccinate and succinylacetone to be the most discriminating biomarkers (area > 0.92).

**Conclusions:**

Age-dependent acylcarnitine correlations may represent metabolic compensation responsive to age-related changes in GHB and GABA. Our study highlights novel biomarkers in SSADHD and expands the metabolic pathophysiology of this rare disorder of GABA metabolism.

## Background

Succinic semialdehyde dehydrogenase (SSADH) deficiency (SSADHD) is a rare disorder on the GABA metabolic pathway. The product of glutamate decarboxylation, GABA is catabolized to succinic acid in a two-enzyme sequence, including generation of succinic semialdehyde (SSA) catalyzed by GABA-aminotransferase and the further oxidation of SSA to succinic acid catalyzed by SSADH [10]. The backbone of GABA thus enters the tricarboxylic acid cycle for further metabolism. SSADHD is frequently referred to as γ-hydroxybutyric aciduria. This is due to the fact that accumulated SSA, in the absence of functional SSADH activity, is converted to γ-hydroxybutyrate (GHB), the latter being a compound with diverse and still poorly understood neuromodulatory roles [13]. In addition to increased GABA and GHB, patients manifest a broad variety of additional metabolic abnormalities, which have recently been summarized [2, 11].

The phenotype of SSADHD encompasses developmental and speech delays, hypotonia, neuropsychiatric morbidity (attention deficit disorder, obsessive compulsive behavior), and seizures in ~ 50% of patients. The metabolic signature includes elevated GHB and GABA in physiological fluids, among other metabolites [11]. The incidence of SSADHD is estimated to be 1:10^6^, but expanding molecular genetic studies are likely to reveal a higher prevalence. To explore the clinical and metabolic evolution of SSADHD, we have recently embarked on an NIH-funded natural history study. Enrolling up to 55 patients (any age), this study encompasses multiple neuroimaging and electrophysiological evaluations over a 5-year period. An important ancillary component of clinical evaluation is the collection of biospecimens yearly, including plasma, white and red cells, DNA and RNA, hair, saliva, stool, urine, fibroblasts, and others. These samples, aliquoted and stored long-term, provide unique opportunities for future analyses as our knowledge of SSADHD evolves, and the potential to correlate biomarkers levels in different matrices with clinical features and disease severity.

Earlier studies in plasma and RBC samples derived from SSADHD patients identified age-dependent negative correlations for both GHB and GABA [10]. GHB in plasma reached a nadir at approximately the age of puberty (~ 13–16 years of age), while the same nadir for GABA occurred well into the 3rd decade of life. DiBacco et al. [6] observed an age-dependent association with worsening of epilepsy, obsessive-compulsive behavior, and sleep disturbances in an older SSADHD cohort. This suggests that declining GABA and GHB levels in peripheral biofluids might represent a metabolic correlate of adulthood symptom severity. Based on this rationale, we examined the hypothesis that other biomarkers of SSADHD (amino acids, acylcarnitines, etc.) would manifest an age-dependent negative correlation as seen for GHB and GABA.

## Results

**Fig. 1:**
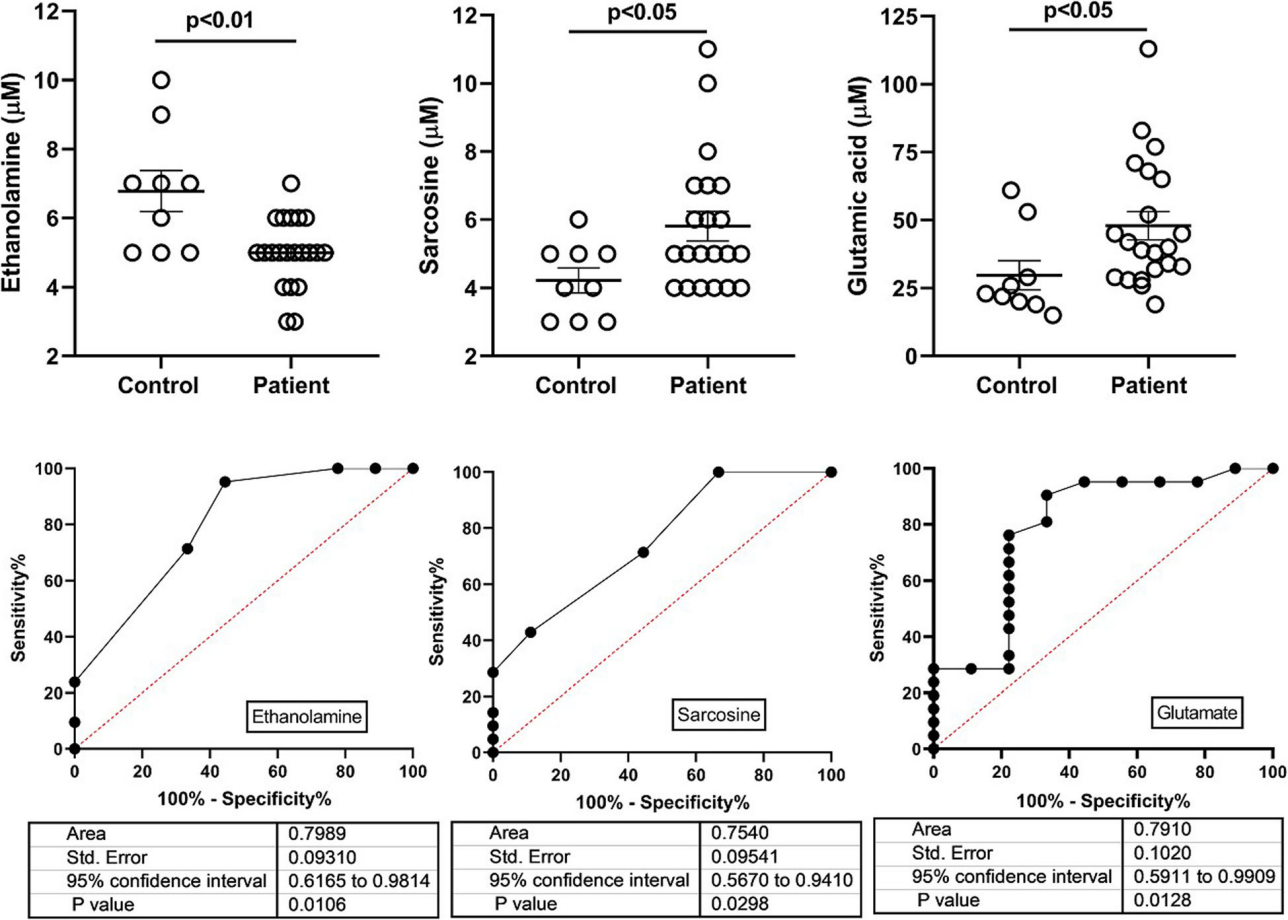
Significant changes in plasma amino acids and corresponding ROC curves for controls and patient cohorts. Data are presented as mean + SEM. Data analysis performed using a two-tailed *t* test

**Fig. 2:**
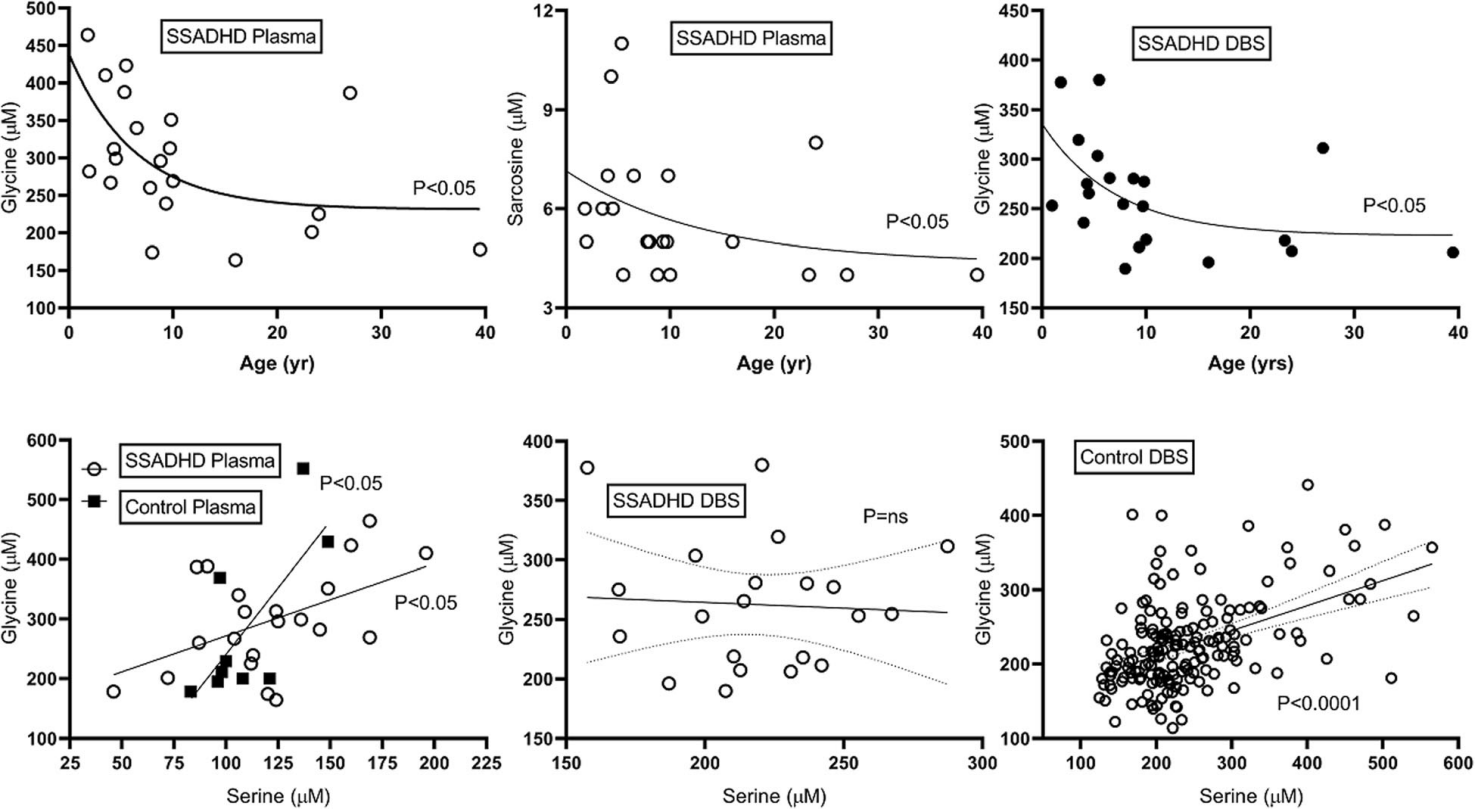
Amino acid correlations with age for patients and controls in plasma and DBS. Statistical analyses employed either the Pearson correlation or the Spearman ranked correlation

**Fig. 3:**
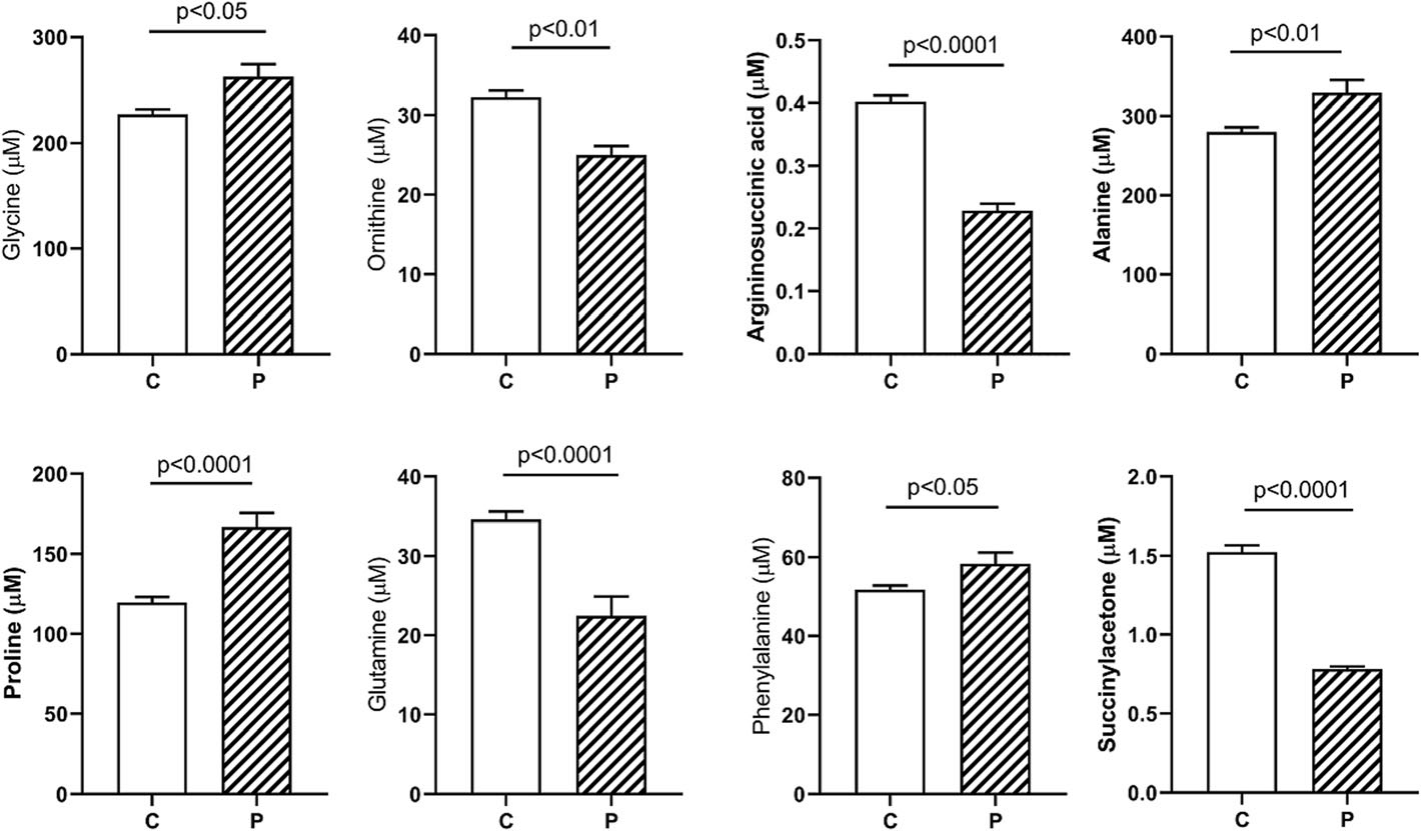
Abnormal amino acids in DBS for controls (C) and patients (P). Data depicted as mean + SEM. Statistical analysis employed a two-tailed *t* test

**Fig. 4:**
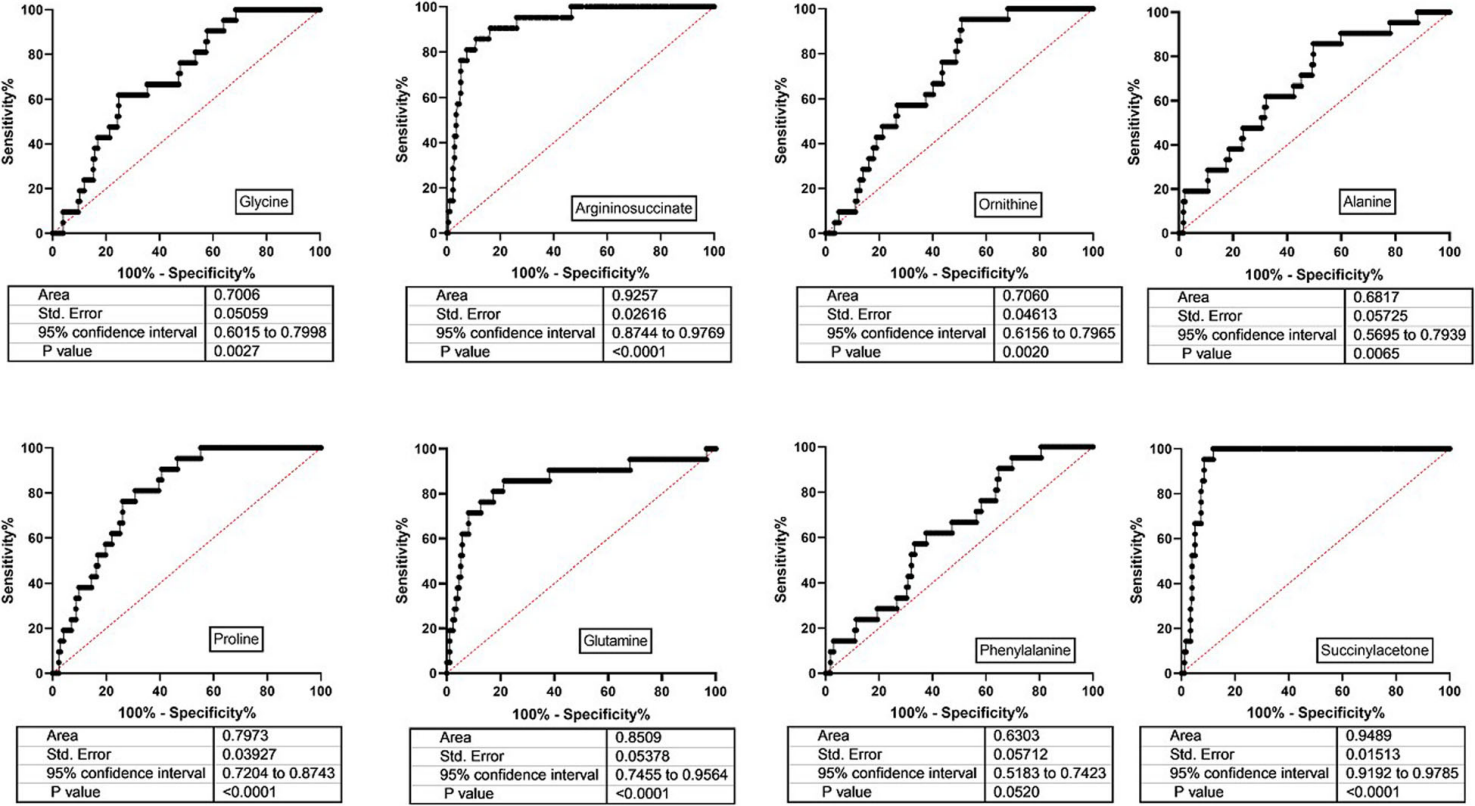
ROC curves corresponding to the amino acid data shown in Fig. 3

Significant elevations of sarcosine (sarc) and glutamate (glu), and a significant decrease in ethanolamine (EA), were demonstrated in SSADHD plasma (Fig. 1). Age-dependent negative correlations were observed for glycine (gly) and sarc in patients, and absent in parallel controls (Fig. 2; control data not shown). The negative correlation for gly in patients was replicated in DBS (Fig. 2; sarc not measured in DBS). An age-dependent negative correlation was observed for serine (ser) in patients that mirrored a comparable age-dependent negative correlation in controls (data not shown). Based on the correlations for gly and ser, we correlated these amino acids in different matrices. In plasma, there were significant positive correlations for both control and SSADHD cohorts (Fig. 2). There was a significant correlation for gly and ser in control DBS, but not in the SSADHD cohort (Fig. 2), likely reflecting the negative age-dependent gly correlation in patients. In patient DBS, elevations of gly, alanine (ala), proline (pro) and phenylalanine were accompanied by significantly decreased ornithine (orn), argininosuccinate (ASA), glutamine (gln) and succinylacetone (Suac) (Fig. 3; Fig. 4 depicts ROC curves corresponding to Fig. 3).

**Fig. 5:**
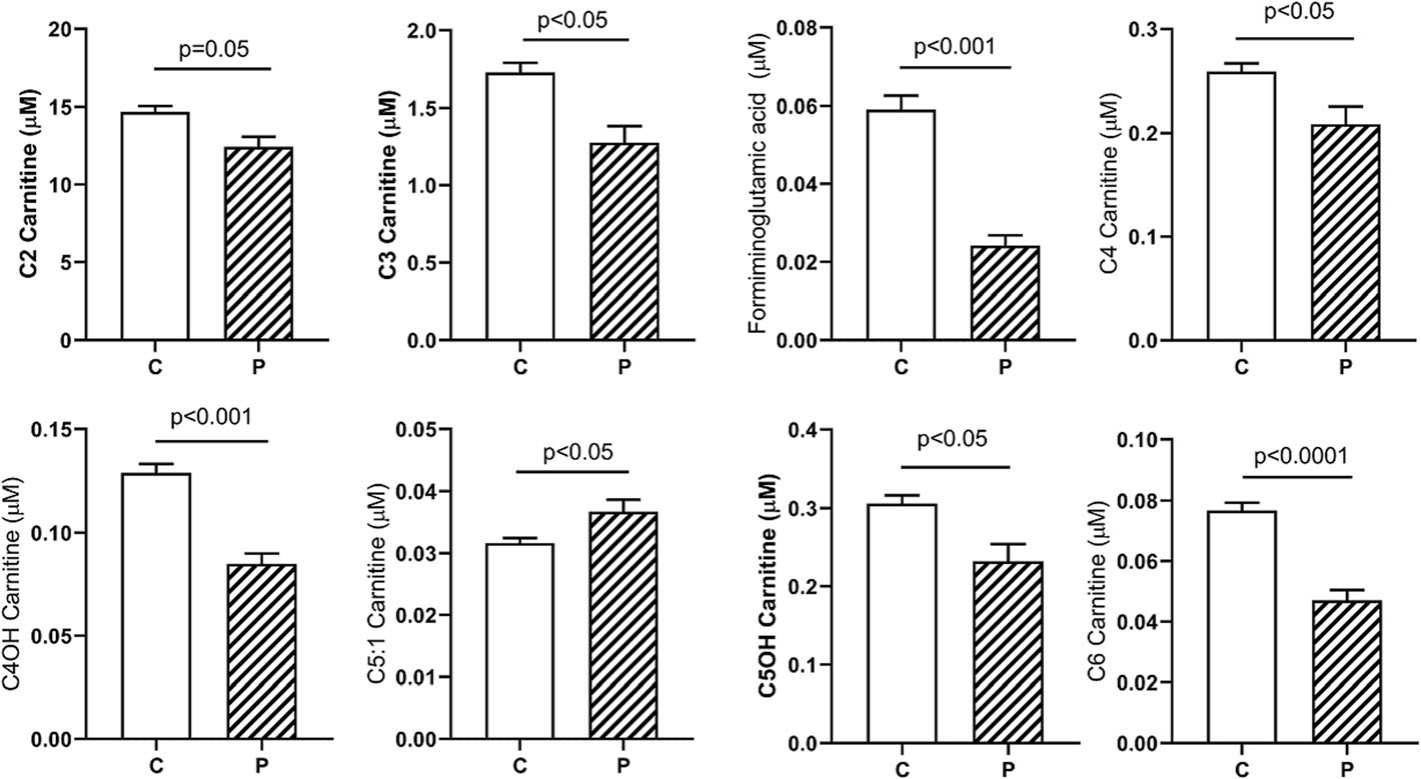
Abnormal short-chain acylcarnitines in DBS for controls (C) and patients (P). Data depicted as mean + SEM. Statistical analysis employed a two-tailed *t* test

**Fig. 6:**
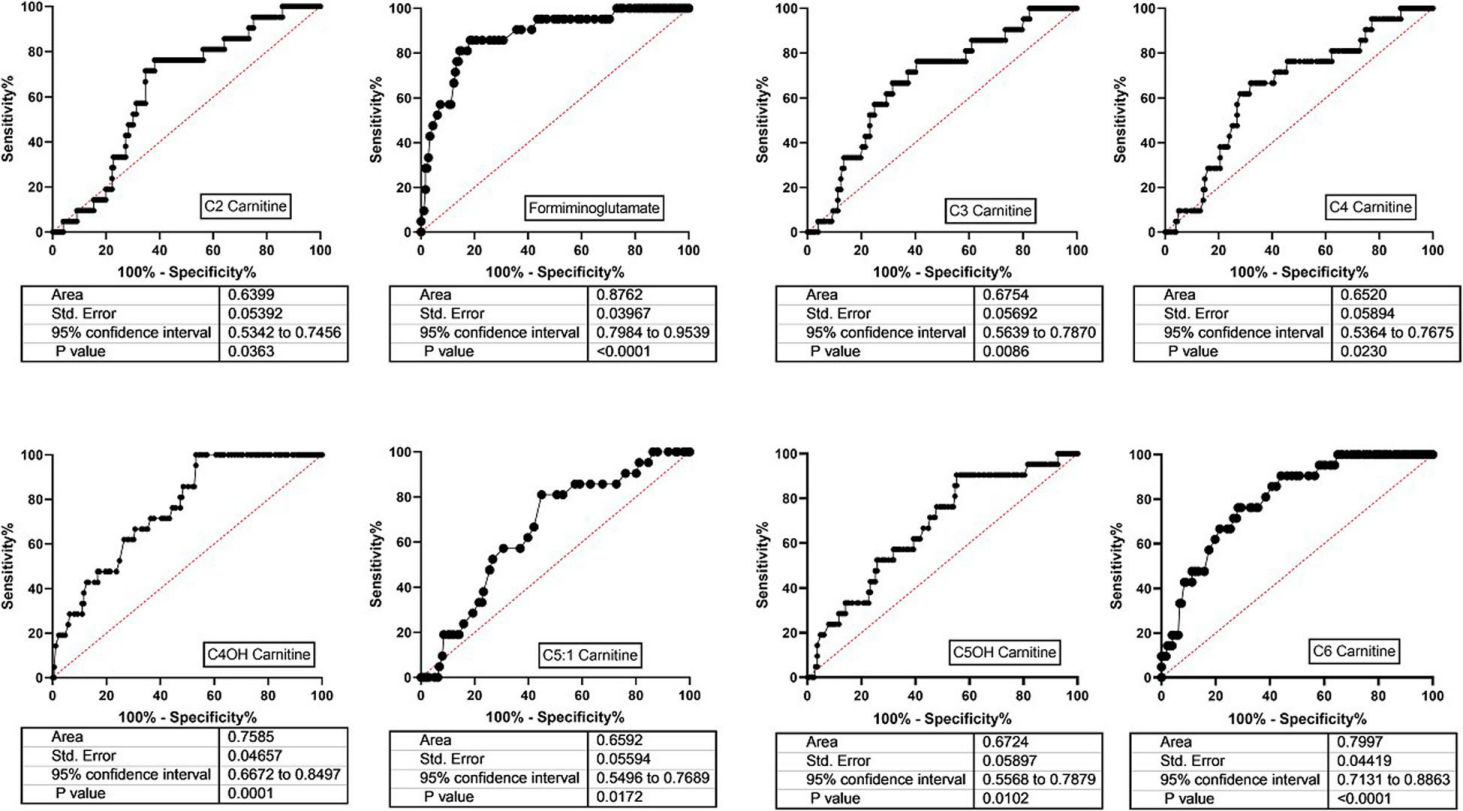
ROC curves corresponding to the acylcarnitine data shown in Fig. 5

Of thirty eight acylcarnitine species quantified, significant alterations were detected in twenty four. Twenty three included significant decreases in patients compared to control, with only C5:1 carnitine showing an elevation (Fig. 5). Decreased short chain acylcarnitines included C2-, C3-, formiminoglutamate (figlu), C4-, C4OH, C5OH and C6 carnitines (Fig. 5; Fig. 6 depicts ROC curves corresponding to Fig. 5). Medium chain acylcarnitines that were significantly decreased in patient DBS included C8-, C8:1, C10- and C10:1 carnitines (Suppl. Fig. 1). In terms of long-chain acylcarnitines, significantly decreased levels in patients included C14-, C14:1, C14:2 and C14OH- carnitines (Suppl. Fig. 2), C16-, C16:1- and C16OH carnitines (Suppl. Fig. 3), and C18-, C18:1-, C18:2-, C18OH and C18:1OH-carnitines (Suppl. Fig. 4; Suppl. Fig. 5 depicts the ROC curves corresponding to Suppl. Fig. 4).

**Fig. 7:**
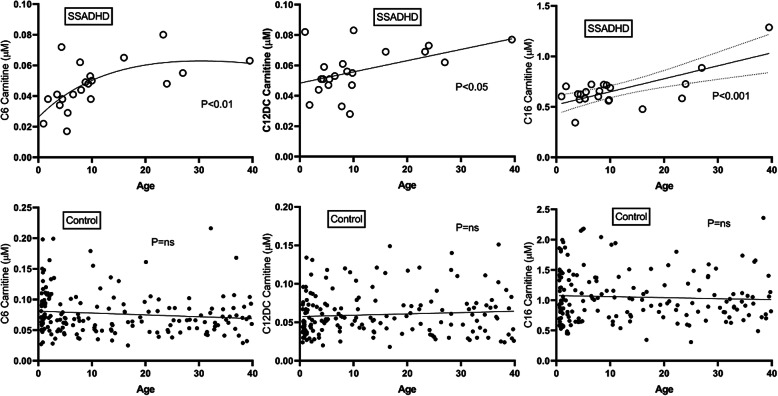
Correlation of C6-, C12DC (dicarboxylic)-, and C16-carnitines with age in DBS for patients and controls. Statistical analyses employed either the Pearson correlation or the Spearman ranked correlation. Abbreviation: ns, not significant

Based on results for gly and sarc, and earlier results for GABA and GHB, correlative analyses were examined as a function of age for all acylcarnitines in both patient and control cohorts. Significant age-related correlations were observed for C3-, C16:1OH- and C18:2-carnitines in patients DBS, but comparable correlations were observed in controls (data not shown). Conversely, significant age-dependent positive correlations were observed for C6-, C12DC-, C16-, C16:1, C18:1 and C18:2OH carnitines in patients which were absent in controls (Fig. 7, Suppl. Fig. 6).

Patients with SSADHD displayed significant alterations in creatinine (crn) and guanidinoacetate (guac) in DBS without concomitant changes in creatine (cre) (Suppl. Fig. 7). Controls demonstrated significant age-related positive correlations for guac (*P* < 0.0001), cre (*P* < 0.05) and crn (P < 0.0001), whereas patients demonstrated this correlation only for crn (P < 0.0001, data not shown). Correlative analyses for all three metabolites were assessed in both cohorts. Controls demonstrated significant age-related positive correlations cre vs crn (P < 0.0001), guac vs. crn (P < 0.0001), but no significant correlation for cre vs. guac (data not shown); conversely, patients demonstrated no correlations for the same comparisons (data not shown), although there was a modest trend for guac vs. crn (*P* = 0.077; data not shown).

## Discussion

Gly and its *N*-methylated precursor, sarc, showed an age-dependent negative correlation in patients, and the gly trend was confirmed in patient plasma and DBS. Like GABA, gly is an inhibitory neurotransmitter, and sarc is an inhibitor of the glycine type I transporter [8, 15]. The nadirs for gly and sarc approximated the same time point (mid-teens, roughly the onset of puberty) as earlier observed for GHB [10]. Although both DBS and plasma demonstrated age-related negative correlations, the glycine level when directly comparing patients and controls was increased only in DBS and not plasma (Fig. 3). Consistent with the age-related nadir for peripheral gly, we detected normal gly in autopsied brain from an adult SSADHD patient (37 years) [11]. Elevated plasma glutamate (glu), the GABA precursor, was demonstrated in patient plasma. Thus, there is now evidence for dysregulation of four neuromodulators (GABA, GHB, glu, gly) in SSADHD biofluids, although we have not documented an age-dependent correlation for glu.

We recently characterized archival newborn screening DBS from patients with SSADHD [4]. Since GHB and GABA are not quantified on any US state newborn screening panel, we sought to identify a metabolic “footprint” suggestive of SSADHD. Using an algorithm measuring the same metabolites as in the current study, informative biomarkers were identified when contrasted with the median and 10th, 25th, 50th, 75th and 90th-centiles of parallel control metabolites. Informative biomarkers (at or below the 1st-centile) included C2-, C3-, C4-, C4OH-carnitines, orn and cre. For post newborn SSADHD DBS, informative biomarkers included C2- and C4OH carnitines, orn, his, and cre. Overall, results for amino acids in the current study (orn, pro, gln (Fig. 3), short-chain acylcarnitines (Fig. 4), and medium-chain acylcarntines (Suppl. Fig. 1)) agreed well with these earlier studies [4]. The finding of significantly decreased ASA, a urea cycle intermediate, is consistent with low orn. Low glutamine (gln) in DBS (Fig. 3) is in-line with multiple studies on SSADHD, including human and animal models, and suggests disruption of the glutamine-glutamate-GABA cycle (GGG [3, 5, 7, 11];). The current study, however, highlighted several novel biomarkers, including pro, suac, and figlu, as well as significant decreases of long-chain acylcarnitines (C14-, C16- and C18- species). Further, when measured in regional extracts of autopsied SSADHD brain ([11]; online supplementary data), long-chain acylcarnitines were consistently below the median and quartile reference ranges, especially in frontal and parietal cortices, pons, and hippocampus.

Twenty three acylcarnitines were significantly decreased and only C5:1 carnitine significantly increased. Considering that GABA degradation impacts the Krebs cycle via entry of succinic acid (end-product of GABA catabolism), as well as removal of α-ketoglutarate (the nitrogen acceptor for transamination of GABA to succinic semialdehyde), it is reasonable that the β-oxidation would be enhanced to provide acetyl-CoA to replenish Krebs cycle function. The C5:1 carnitine species arises from the metabolism of leucine, which converts β-methylcrotonyl-CoA to acetoacetate and aceytyl-CoA, and tiglyl-CoA to propionyl-CoA, respectively. GHB itself, or a GHB metabolite (3-oxo-4-hydroxybutyrate, 4-hydroxycrotonate) may inhibit both metabolic sequences based upon structural similarities, either as free acid salts or the associated coenzyme A ester [2]. This would explain elevated C5:1 carnitine, supported by the concomitant decrease of C3-carnitine (propionyl-carnitine), the end-product of isoleucine metabolism in which tiglyl-CoA is an intermediate.

We identified guac and crn as biomarkers of SSADHD in DBS (Suppl. Fig. 7). Low crn is consistent with low crn identified in previous SSADHD post-newborn DBS assessing the potential for newborn screening of SSADHD [4]. Elevation of guac has previously been reported in biofluids of patients and tissue extracts of the murine model [9], and regional brain extracts of autopsied SSADHD brain also revealed elevated guac [11]. The spontaneous non-enzymatic interconversion of cre to crn may offer an explanation of why we did not observe changes in cre levels in the currentstudy.

**Fig. 8:**
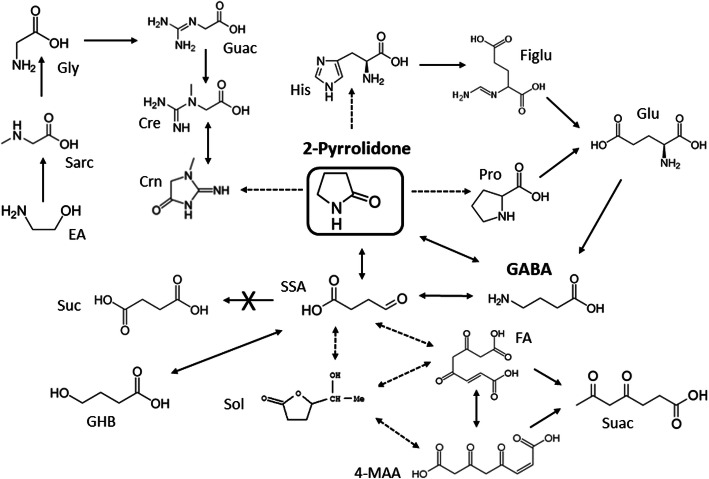
Potential mechanisms leading to biomarker abnormalities in SSADHD patients. The pathway of GABA metabolism is shown, with X indicating the site of the block in patients with SSADHD. This results in accumulation of GHB, in addition to GABA and succinic semialdehyde (SSA). Under physiological conditions, GABA can undergo lactonization to form the internal γ-lactam, 2-pyrrolidone (2-pyr [12];). Structural similarities of 2-pyr with the imidazole/pyrrolidine structures of crn, pro and his may associate with alterations in EA, sarc, gly, guac, crn, figlu and pro. Brown et al. [1] first proposed that 4,5-dihydroxyhexanoate (4,5-DHHA; also solerole (sol)) derived from accumulated SSA and an “active” 2-carbon fragment (acetyl-CoA, or the *acyl-* moiety of pyruvic acid). 4,5-DHHA can undergo internal lactonization to at least two species in relation to its vicinal *hydroxyl-* groups, one of which is solerole. A potential combination of accumulated 4,5-DHHA, sol, SSA or GHB may result in interference with the distal portion of tyrosine metabolism at the level of 4-maleylacetoacetate (4-MAA) and/or fumarylacetoacetate (FA), resulting in decreased Suac. Dashed lines indicate proposed pathways of interference/inhibition

Our natural history study of SSADHD, with enrollment of patients of any age, offers the opportunity for multiple age-related metabolic correlations. Along those lines, we evaluated age-dependent correlations for all metabolites characterized. In addition to negative correlations for gly and sarc, we found age-dependent positive correlations ((C6-, C12DC-, C16-, C16:1-, C18:1-, C18:2OH-carnitines) in the SSADHD cohort that were absent in controls. Whether these correlations represent compensatory changes responsive to those for gly, sarc, GHB, and GABA remains to be evaluated. On the other hand, one metabolite seemingly pathognomonic for SSADHD is the six-carbon 4,5-dihydroxyhexanoate (DHHA, or solerol (sol) when lactonized; Fig. 8 [1, 11];). Metabolic sequences involved in the metabolism/accretion of those acylcarnitines for which age-related positive correlations in patients were identified may be susceptible to inhibition by 4,5-DHHA (Fig. 8) but this too requires further study.

One limitation of the current study is the inability to control for medications in our patient population. Symptomatic interventions in this population usually address neuropsychiatric morbidity (lamotrigine, zonisamide, topiramate, benzodiazepines as needed) but the use of valproate is generally contraindicated. The impact of these medications on the biochemical disturbances observed is not fully known. Our studies confirm that, in addition to disruptions of the GGG cycle, the GABA shunt, and oxidative phosphorylation, SSADHD appears to significantly alter β-oxidation, urea and creatine cycles, the sarcosine pathway, as well as multiple amino acid pathways [2, 5, 14]. GABA can cyclize to 2-pyrrolidone, its internal γ-lactam, a recently described biomarker for GABA-transaminase deficiency [12]. We postulate that 2-pyrrolidone (2-pyr) may provide the link between some of the novel biomarkers identified in the current study, including crn, pro, and figlu (Fig. 8). The structural similarity of 2-pyr to the imidazole/pyrrolidine rings of his, crn and pro, may lead to interference with those metabolic sequences. Similarly, we speculate that interference of tyrosine metabolism at the level of 4-maleylacetoacetate /fumarylacetoacetate leads to decreased levels of suac in DBS (Fig. 8), perhaps by accumulated 4,5-DHHA, SSA, or GHB itself.

## Conclusions

We have highlighted a number of novel biomarkers in SSADHD, including crn, pro, figlu, sarc, suac, ASA, and EA, as well as age-dependent correlations for sarc and gly and several acylcarnitine species that appear unique for SSADHD. Of those, we found the most discriminating biomarkers to be figlu (area > 0.87) and suac/ASA, with areas > 0.92. We will correlate the age-dependent fluctuation of these biomarkers, along with GABA and GHB, to clinical outcomes (neuroimaging, electrophysiology) in our ongoing natural history study of SSADHD. The objective is to expand insight into the evolving metabolic and clinical pathophysiology of SSADHD, and potentially identify selective metabolic markers that provide predictive insight into the non-specific course of the disease.

## Methods

DBS cards were obtained from twenty-one unique patients with SSADHD (age range 0.95–39.5 years (mean, 11 years; median 8 years); 12 F, 9 M). The samples were obtained at intake in year 01 of our natural history study. SSADHD was previously confirmed through a combination of GHB measurement (urine, blood, DBS), molecular genetic analyses, or assay of SSADH activity in white cells [4]. Parallel control DBS cards were derived from 9 unique individuals (age range 8.4–34.8 years (mean, 18.1 year; median, 16.8 years); 5 F, 4 M. DBS were obtained using standard finger lance and blood collected onto 903 five spot blood cards (Eastern Business Cards, Greenville, South Carolina). EDTA plasma was obtained from the same patient/control cohorts. Archival reference control data was available (*n* = 171; age range 0.5–39.9 years (median, 7.7 years); 80 F, 91 M) for comparison purposes (https://clir.mayo.edu/).

Amino acids, acylcarnitines, creatine, creatinine, guanidinoacetate, and succinylacetone were quantified in DBS using tandem mass spectrometry as previously described [16]. Amino acids in plasma were quantified by MassTrak analysis as described [17]. Grouped cohorts (patient, control; mean +/- SEM) were evaluated using a two-tailed Student’s *t* test. As this study was exploratory with regard to age, we chose to use univariate statistical analyses without correction for the *p* value. ROC curves were constructed for all binary comparisons (patient vs. control cohorts). Correlation with age was assessed using the Pearson coefficient or Spearman’s ranked test. Significance was set at *p* < 0.05. Data was evaluated using GraphPad Prism version 8.0.

## Supplementary information

**Additional file 1 Figure S1**. Abnormal medium-chain acylcarnitines and ROC curves in DBS of controls (C) and patients (P). Data depicted as mean + SEM. Statistical analysis employed a two-tailed *t* test.

**Additional file 2 Figure S2**. Abnormal C14 acylcarnitines and ROC curves in DBS of controls (C) and patients (P). Data depicted as mean + SEM. Statistical analysis employed a two-tailed *t* test.

**Additional file 3 Figure S3**. Abnormal C16 acylcarnitines and ROC curves in DBS of controls (C) and patients (P). Data depicted as mean + SEM. Statistical analysis employed a two-tailed *t* test.

**Additional file 4 Figure S4**. Abnormal C18 acylcarnitines in DBS of controls (C) and patients (P). Data depicted as mean + SEM. Statistical analysis employed a two-tailed *t* test.

**Additional file 5 Figure S5**. ROC curves corresponding to the long-chain acylcarnitine data shown in Suppl. Fig. 4.

**Additional file 6 Figure S6**. Correlation of C16:1-, C18:1, and C18:2OH-carnitines with age in DBS for patients and controls. Statistical analyses employed either the Pearson correlation coefficient or the Spearman ranked test. Abbreviation: ns, not significant.

**Additional file 7 Figure S7**. Concentration of crn and guac in DBS of controls (C) and patients (P). Data are presented as mean + SEM. Data analysis performed using a two-tailed *t* test.

## Data Availability

The datasets used and/or analyzed during the current study are available from the corresponding author upon reasonable request.

## Abbreviations

GABA: γ-aminobutyrate
GHB: γ-hydroxybutyrate
SSA: Succinic semialdehyde
4,5-DHHA: 4,5-dihydroxyhexanoate (when lactonized, 4,5-dihydroxyhexanoate γ-lactone; solerole (Sol))
Ala: Alanine
Glu: Glutamate
Gln: Glutamine
Pro: Proline
His: Histidine
Orn: Ornithine
Gly: Glycine
Ser: Serine
Sarc: Sarcosine
Suc: Succinate
EA: Ethanolamine
Guac: Guanidinoacetate
Cre: Creatine
Crn: Creatinine
Suac: Succinylacetone
Figlu: Formaminoglutamate
GGG: Glutamine-glutamate-GABA cycle
2-pyr: 2-pyrrolidone (γ-lactam of GABA)
CoA: Coenzyme A
FA: Fumarylacetoacetate
4-MAA: 4-maleylacetoacetate
ROC curve: Receiver operating characteristic curve
