# Supplementary figures and images for: Novel biomarkers and age-related metabolite correlations in plasma and dried blood spots from patients with succinic semialdehyde dehydrogenase deficiency
Source: Orphanet J Rare Dis. 2020 Sep 23;15:261. doi: 10.1186/s13023-020-01522-5 (PMC7510106; doi:10.1186/s13023-020-01522-5)

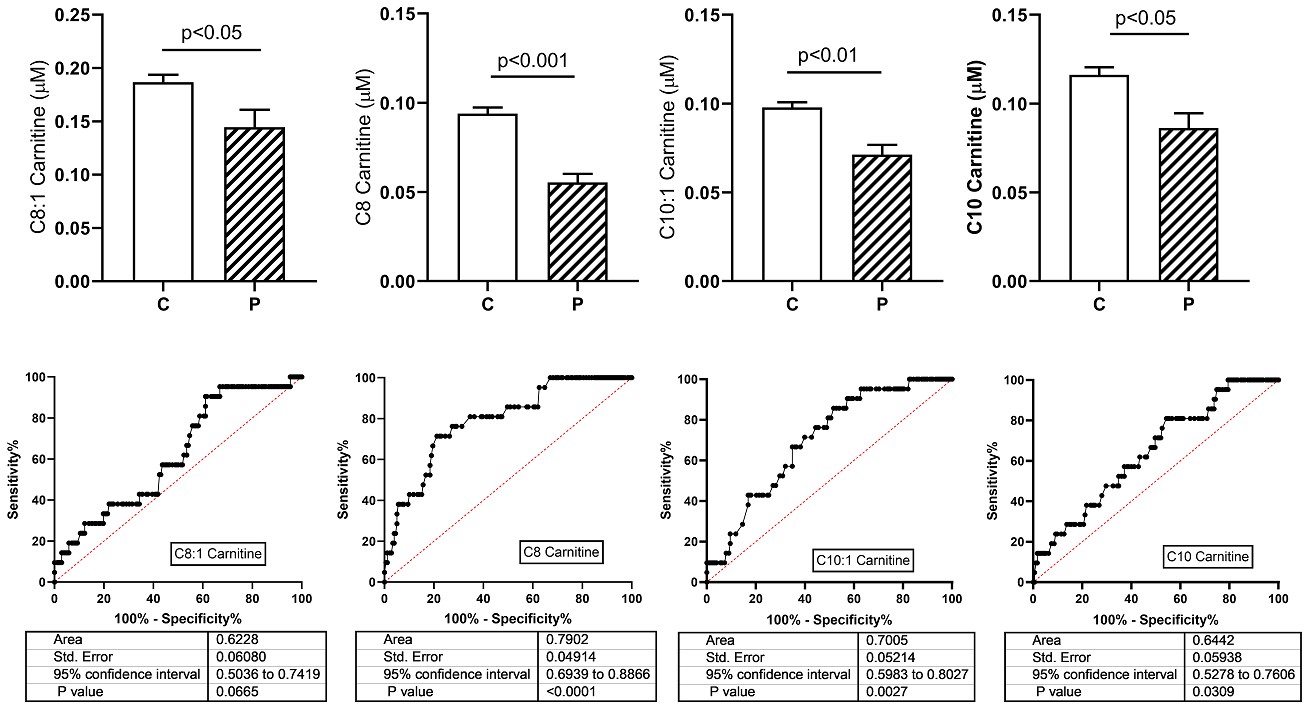

Supplement: Supplementary file 1 — Additional file 1 Figure S1. Abnormal medium-chain acylcarnitines and ROC curves in DBS of controls (C) and patients (P). Data depicted as mean + SEM. Statistical analysis employed a two-tailed t test. [file 13023_2020_1522_MOESM1_ESM.jpg]

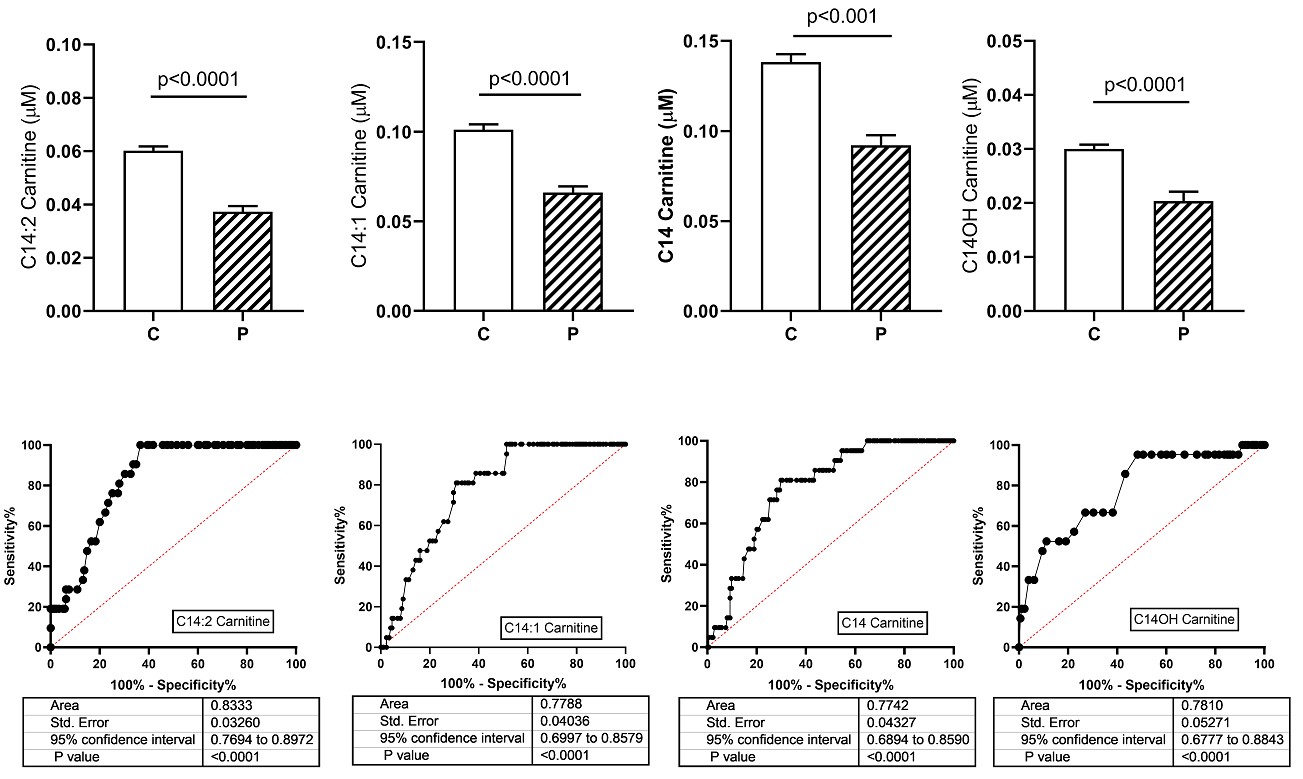

Supplement: Supplementary file 2 — Additional file 2 Figure S2. Abnormal C14 acylcarnitines and ROC curves in DBS of controls (C) and patients (P). Data depicted as mean + SEM. Statistical analysis employed a two-tailed t test. [file 13023_2020_1522_MOESM2_ESM.jpg]

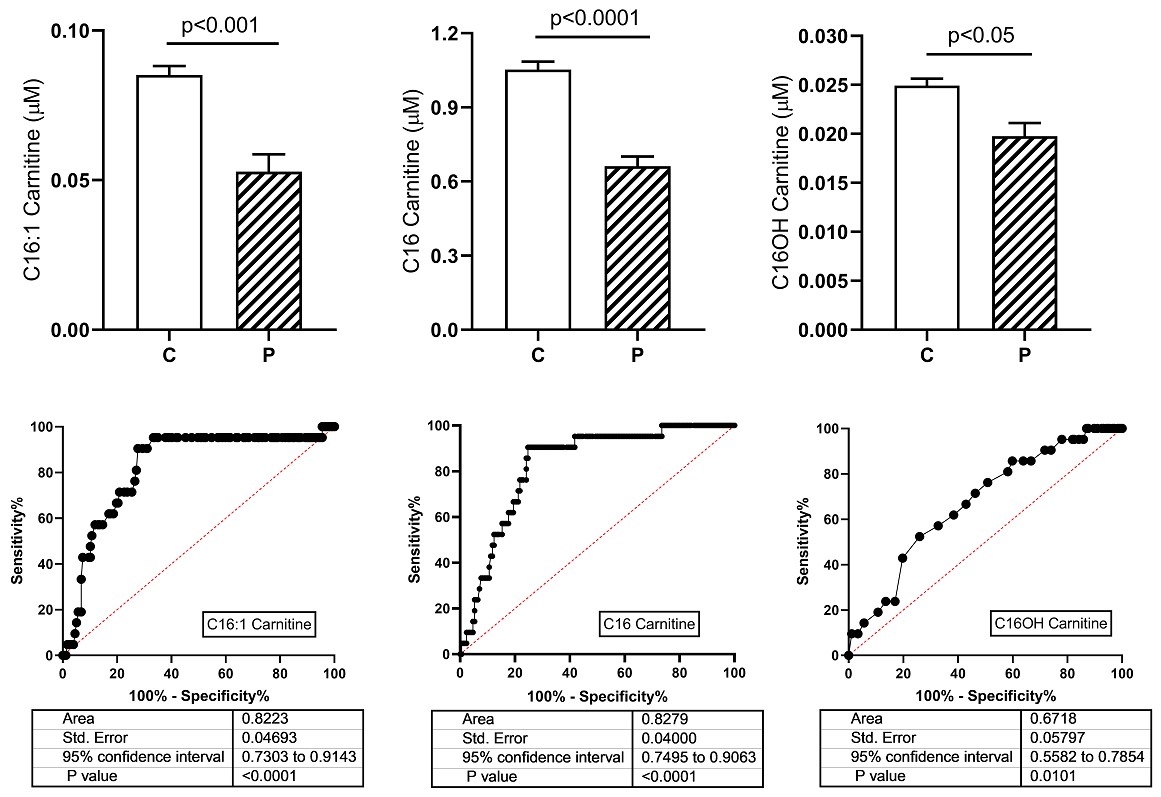

Supplement: Supplementary file 3 — Additional file 3 Figure S3. Abnormal C16 acylcarnitines and ROC curves in DBS of controls (C) and patients (P). Data depicted as mean + SEM. Statistical analysis employed a two-tailed t test. [file 13023_2020_1522_MOESM3_ESM.jpg]

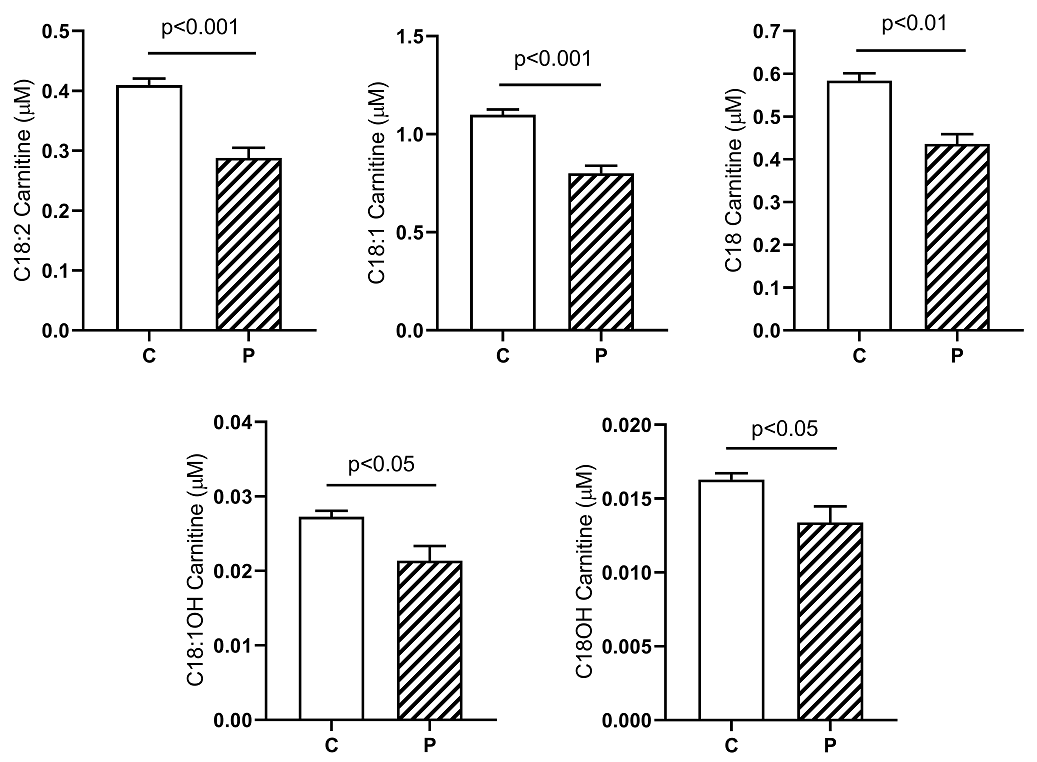

Supplement: Supplementary file 4 — Additional file 4 Figure S4. Abnormal C18 acylcarnitines in DBS of controls (C) and patients (P). Data depicted as mean + SEM. Statistical analysis employed a two-tailed t test. [file 13023_2020_1522_MOESM4_ESM.tif]

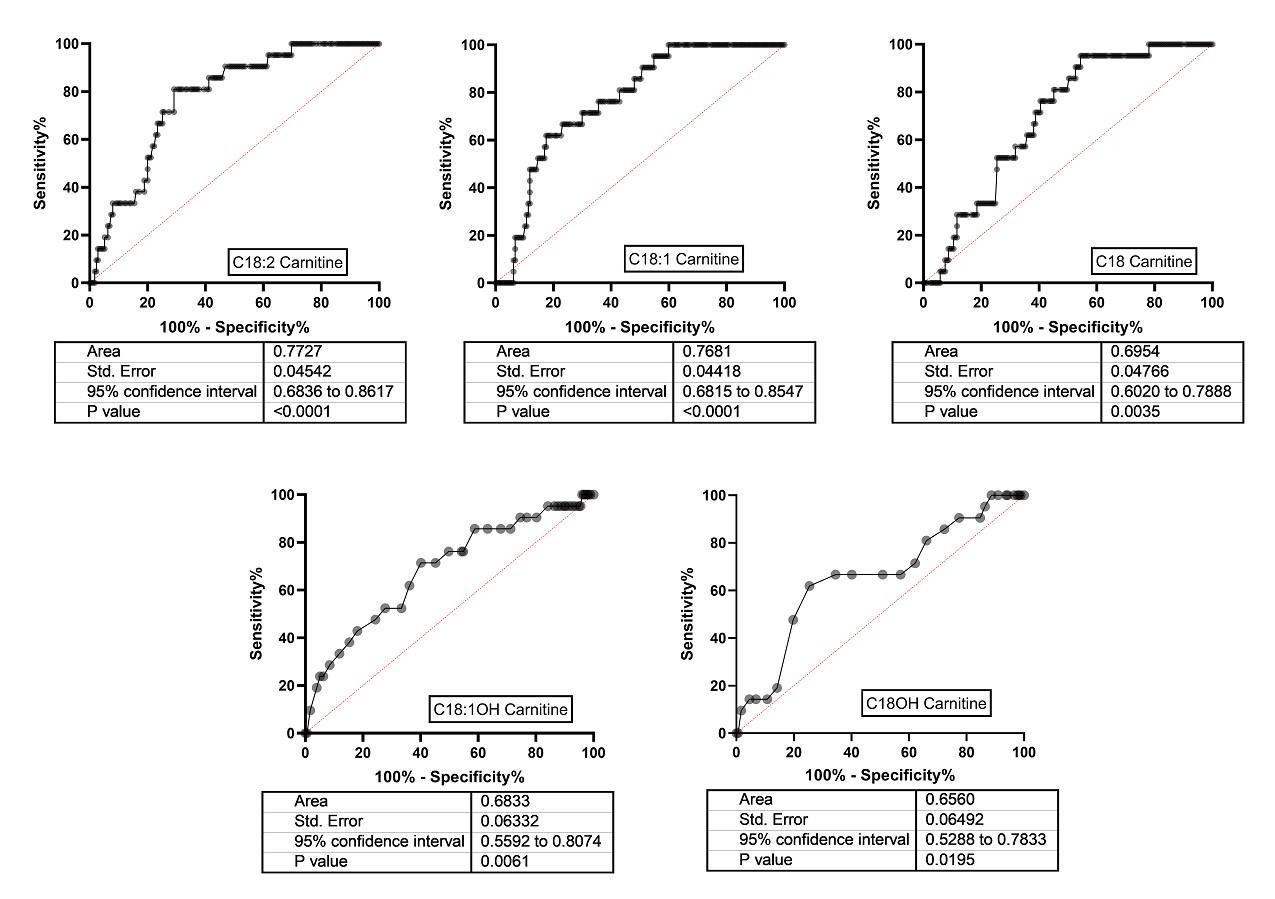

Supplement: Supplementary file 5 — Additional file 5 Figure S5. ROC curves corresponding to the long-chain acylcarnitine data shown in Suppl. Fig. 4. [file 13023_2020_1522_MOESM5_ESM.jpg]

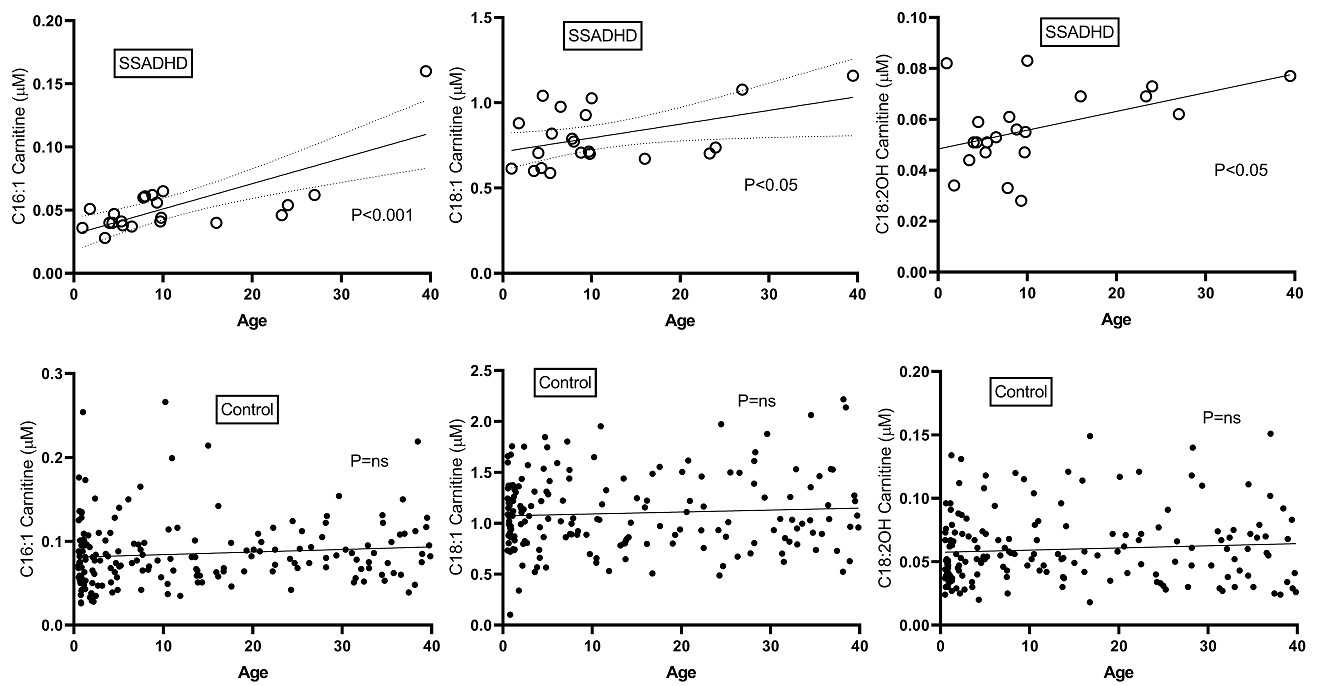

Supplement: Supplementary file 6 — Additional file 6 Figure S6. Correlation of C16:1-, C18:1, and C18:2OH-carnitines with age in DBS for patients and controls. Statistical analyses employed either the Pearson correlation coefficient or the Spearman ranked test. Abbreviation: ns, not significant. [file 13023_2020_1522_MOESM6_ESM.jpg]

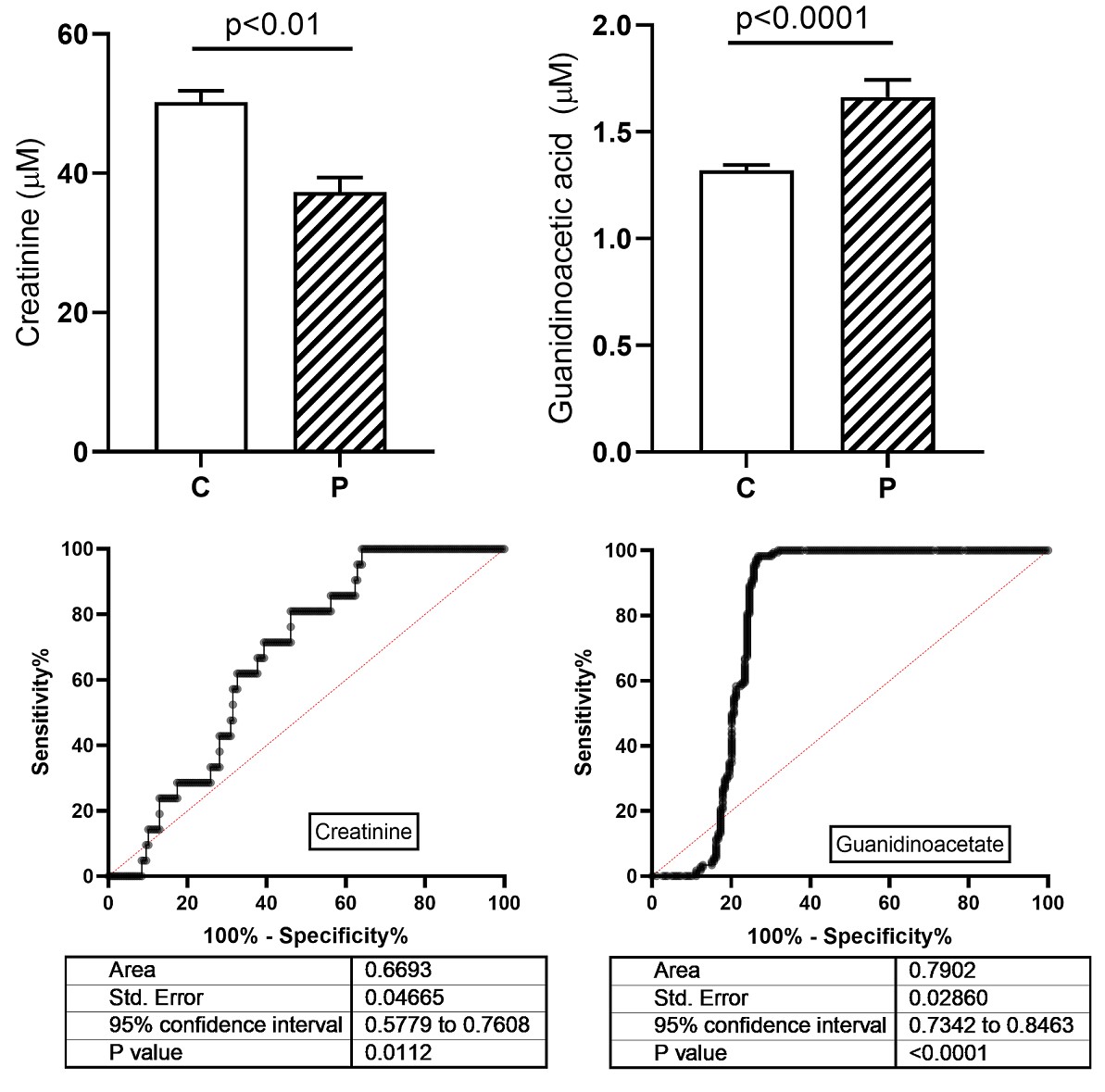

Supplement: Supplementary file 7 — Additional file 7 Figure S7. Concentration of crn and guac in DBS of controls (C) and patients (P). Data are presented as mean + SEM. Data analysis performed using a two-tailed t test. [file 13023_2020_1522_MOESM7_ESM.jpg]
